# Neurobehavioral effects of general anesthesia and cochlear implantation on hearing‐impaired infants: A prospective observational cohort study

**DOI:** 10.1002/brb3.3216

**Published:** 2023-08-13

**Authors:** Li Ma, Yue Yu, Xuhui Zhou, Jinya Shi, Nanyang Le, Yudan Liang, Jingjie Li, Hong Jiang

**Affiliations:** ^1^ Department of Anaesthesiology Shanghai Ninth People's Hospital Shanghai Jiao Tong University School of Medicine Shanghai China

**Keywords:** cochlear implantation, general anesthesia, infant, prolonged anesthetic exposure

## Abstract

**Introduction:**

The potential adverse effects of prolonged exposure to anesthetics in pediatric patients with severe‐to‐profound sensorineural hearing loss remain unclear. This study aimed to examine whether early bilateral cochlear implantation involving long‐duration anesthetic exposure caused greater developmental impairment than that with unilateral cochlear implantation.

**Methods:**

This prospective observational study included normally developing infants with bilateral severe‐to‐profound sensorineural hearing loss aged 6 months to 2 years who were candidates for unilateral/bilateral cochlear implantation surgery. Baseline (T0), 6‐month (T1), and 1‐year (T2) Gesell Scale scores were measured. The outcomes included fine motor, adaptability, gross motor, language, and social skills scale 6 and 12 months postoperatively.

**Result:**

The 90 enrolled children with bilateral severe‐to‐profound sensorineural hearing loss (unilateral n = 43; bilateral n = 47) had a younger bilateral group (11.00 ± 3.66 vs. 15.63 ± 6.99 months, *p* < .001). Anesthesia duration was longer in the bilateral group (271.57 ± 36.09 vs. 148.81 ± 25.60 min, *p* < .001). Gross motor, fine motor, adaptability, and language scores improved in both groups, and no significant between‐group differences occurred in the fine motor scale at T1 and T2. Language developmental quotients improved significantly in the bilateral group compared with the unilateral group at T1 (mean differences: 25.07 ± 4.37 vs. 10.88 ± 4.61, *p* < .001) and T2 (mean differences: 34.98 ± 5.94 vs. 15.28 ± 6.55, *p* < .001). Stepwise regression revealed that gross motor, adaptability, language, and social skill developmental quotients at T1 were positively correlated with those at T0. Gross motor, fine motor, and social skill developmental quotients at T2 were negatively correlated with age at operation. Language developmental quotients were positively correlated with T0 values (*p* < .001) and in the bilateral group (*p* < .001) at T1 and T2.

**Conclusions:**

When evaluating young children with bilateral severe‐to‐profound sensorineural hearing loss, despite longer exposures to general anesthesia, bilateral cochlear implantations were associated with more improvement in language scores and no differences in other skills compared with those with only unilateral implantation.

## Graphical abstract

Prolonged anesthetics exposure does not cause developmental impairment in infants undergoing cochlear implantation using Gesell Development Diagnosis Scale evaluation. In addition, compared with unilateral cochlear implantation, bilateral cochlear implantation in early life significantly improves language ability and promotes the development of adaptability.

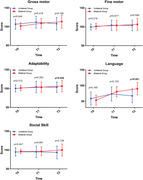

## 1 INTRODUCTION

Annually, millions of children undergo general anesthesia for surgical, procedural, and diagnostic purposes. However, there has been recent growing concern regarding the causal relationship between early‐life general anesthetic exposure and subsequent long‐term neurodevelopmental abnormalities. Although preclinical studies have demonstrated that exposure to anesthetic agents is associated with altered brain development in immature animals, including non‐human primates (Jevtovic‐Todorovic, 2018; Vutskits & Xie, 2016), human observational studies on anesthetic‐induced neurotoxicity have applied heterogeneous methodologies and reported inconsistent findings (Walkden et al., 2019). Among available studies, significant heterogeneity exists in the type of surgical procedure, number of exposures, comparators, methods used to adjust for potential confounding (if any), and the outcomes examined (Ing et al., 2022). Infants and young children who undergo multiple or prolonged anesthetic exposures are more likely to have a higher rate of comorbid conditions, including prematurity (Ing et al., 2022), low birth weight (Shi et al., 2018), and higher American Society of Anesthesiologists status (Bartels et al., 2018).

Although there have been many studies on anesthetic‐induced neurotoxicity in young animals (Alvarado et al., 2017; Raper et al., 2017), addressing unmeasured confounding and bias may be more complicated in clinical settings. Randomizing children with a similar disease condition to not receiving anesthesia for surgery is difficult or impossible. Thus, some clinical studies selected twins or siblings to attempt to eliminate genetic confounding and environmental factors, such as uterine environment, parental education, parenting style, home/family environment, neighborhood, educational, and socioeconomic factors (Walkden et al., 2019). A sibling‐matched cohort study reported that healthy children with a single anesthesia exposure before the age of 36 months compared with those with no anesthesia exposure had no significant differences in intelligence quotient scores in later childhood (Sun et al., 2016). Additionally, exposure to general anesthesia before primary school entry did not increase the risk of adverse child development outcomes in another matched sibling pairs study (O'leary et al., 2019). Therefore, further studies with standardized methodologies comparing the outcomes of different exposure durations with adjustment of individual differences are warranted to yield strong findings.

Cochlear implantation has become a standard intervention for infants with bilateral severe‐to‐profound sensorineural hearing loss (SNHL). Since early interventions can minimize the negative long‐term effects of infants with SNHL on communication and quality of life, the minimum eligibility age for implantation has been reduced from 12 to 9 months by the United States Food and Drug Administration (Warner‐Czyz et al., 2022). In China, the eligible age for cochlear implantation candidacy was 12 months, as first published in 2003 (Society of Otolaryngology C M A, 2003); however, this was not rigidly adhered to by clinics. This was subsequently updated in 2013 (Guideline of cochlear implant, 2014) to “as early as possible within the premise of safety.” The otolaryngologists have reported that children with SNHL will benefit more from bilateral cochlear implantation than from unilateral cochlear implantation, including phonological processing skills, language‐based skills, and auditory‐linguistic processing levels in complex listening conditions (Lee, 2021; Lee & Sim, 2020; Yıldırım Gökay & Yücel, 2021). However, currently, in China, the cost of a single cochlear implant could be covered through medical insurance, local government projects, private donations, or charities for most of these children. Conversely, a second cochlear implant might be unavailable for many children with bilateral profound SNHL (Chen et al., 2020). Among them, a few parents choose to monitor the development of communication skills with hearing aids closely or have the potential to perform better with cochlear implants later. This decision‐making significantly depends on economic factors, parental cognition, hearing aids’ effectiveness, and the children's medical comorbidities. However, it also includes a few concerns about younger children's prolonged exposure to anesthetics (FDA Drug Safety Communication, 2016). The duration of operation and anesthesia in bilateral cochlear implantation is longer. Therefore, more attention should be paid to the possible effects of prolonged anesthesia on cognitive function.

As a reliable assessment of motor, adaptability, language, and social‐emotional domains, the Gesell Development Diagnosis Scale (GDDS) has been used to evaluate early development in children with SNHL aged from 4 weeks to 3 years and to predict subsequent cognitive‐developmental trajectories (Meinzen‐Derr et al., 2017). Several clinical studies have demonstrated that assessment using the GDDS is practical and provides fairly robust indicators of outcomes (Meinzen‐Derr et al., 2017; Yang et al., 2017). In our previous retrospective exploratory study, 17 infants aged <3 years underwent bilateral cochlear implantation involving >4 h of anesthesia, with a prolonged anesthesia duration being defined as >3 h. We observed a significant postoperative reduction in fine motor abilities on the GDDS at 6 months postoperatively among participants who received total inhalation anesthesia with sevoflurane rather than propofol (Zhang et al., 2022). Additionally, we previously showed that impaired fine motor control skills and cognitive functions in mice were associated with multiple early‐life exposures to sevoflurane anesthesia. In clinical practice, anesthesiologists prefer using a mixed‐agent general anesthetic regimen to reduce the dosage of a single anesthetic.

Most children with congenital SNHL undergo cochlear implantation before the period of 2–3 years of age. In humans, this stage is reported as a “vulnerable time window” (Clausen et al., 2017; Morriss et al., 2014; Walters & Paule, 2017). The surgery duration of bilateral cochlear implantation, which is almost twice that of unilateral cochlear implantation, is usually over 3 h, which is defined as a “prolonged anesthesia duration” by the United States Food and Drug Administration (Gesell, 1932). Thus, we aimed to investigate the long‐term postoperative effects of bilateral cochlear implantation and unilateral cochlear implantation under general anesthesia on the development of infants with SNHL, with no risk of confounding by indication and a maximization of external validity, in this observational study.

## 2 MATERIALS AND METHODS

### 2.1 Ethical approval

This study was approved by the Human Research Ethical Committee of Shanghai Ninth People's Hospital, Shanghai JiaoTong University of Medicine, P. R. China (SH9H‐2019‐T19‐2), and registered at ClinicalTrials.gov (NCT04255485). Written informed consent was obtained from the patients’ parents or guardians before recruitment. This study was conducted in compliance with the Declaration of Helsinki for Medical Research. This study was reported according to the Strengthening the Reporting of Observational Studies in Epidemiology guidelines.

### 2.2 Participants

Between May 2019 and March 2021, we initially included 96 American Society of Anesthesiologists Physical Status I–II pediatric patients with bilateral SNHL (age: 6 months to 2 years) who underwent unilateral or bilateral electronic cochlear implantation under general anesthesia, as well as preoperative GDDS evaluation of their developmental level. The exclusion criteria were as follows: (i) parents or guardians refusing to sign the informed consent form; (ii) developmental quotient (DQ) of adaptability, fine motor, or gross motor skills of preoperative GDDS evaluation <86; (iii) heart, lung, or nervous system diseases; (iv) severe liver or kidney dysfunction; (v) history of premature birth, fetal distress, hypoxia, or jaundice; (vi) allergy to eggs, milk, or known anesthetics; and (vii) family history of malignant hyperthermia. We excluded children with developmental abnormalities unrelated to auditory abnormalities (GDDS evaluation <86) to avoid possible confounders. Children diagnosed with developmental delay or suspicious of delay (DQ < 86) might seek etiology or treatment during the follow‐up period. These medical interventions may impact research observations.

### 2.3 GDDS evaluation

After identifying a candidate for cochlear implantation, trained clinicians evaluated the patient using the GGDS in the hospital preoperatively. The GDDS comprises the following five domain subscales: adaptability, fine motor, gross motor, language, and social skills. The DQ was calculated as follows: (development age/actual age) × 100. Normal neurological development and developmental delay were defined as DQ ≥86 and DQ ≤75, respectively, while 76≤DQ≤85 was considered suspicious (Gesell, 1932). We used the Chinese version of GDDS, a domestic standardization revision validated and modified by the Beijing Mental Development Cooperative Group in 1985 (Gesell developmental diagnosis scale, 1985), which has been commonly used in pediatric clinical practice in China. It has been clinically used to diagnose developmental delay in infants (Huo et al., 2011; Liu et al., 2016).

The patients were reassessed using the GGDS at 6 months (T1) and 1 year (T2) after the initial activation of the cochlear implant device, with calculation of the DQ of each domain according to the participants’ actual age. The follow‐up windows were set at 6 months ± 30 days (T1) and 1 year ± 45 days (T2) after cochlear device activation.

### 2.4 Variables

Based on our previous study, we chose the changes in the fine motor scale in the GDDS 6 months postoperatively as our primary outcome. The secondary outcomes included the fine motor scale in the GDDS after 12 months and all other scales in the GDDS.

### 2.5 Procedures

General anesthesia was induced using midazolam (0.05−0.1 mg/kg), fentanyl (2−4 μg/kg), propofol (1.5−2.5 mg/kg), and rocuronium (0.6−0.9 mg/kg). After oral intubation, mechanical ventilation was maintained for the duration of the surgery, with the ventilator (Avance CS2; GE Health Care) parameters set at a tidal volume of 8−10 mL/kg and frequency of 18−22 times/min to maintain the oxygen saturation >95%. Combined anesthesia with sevoflurane (1.5−2.0 Vol%) inhalation as well as propofol (2−10 mg·kg^−1^·h^−1^) and remifentanil (0.01−0.2 μg·kg^−1^·min^−1^) intravenous maintenance were used to stabilize the anesthetic effect and maintain the bispectral index ranging from 40 to 60.

### 2.6 Study size

In our previous studies, we found a positive reduction in fine motor abilities 6 months postoperatively in children who received early anesthesia during bilateral cochlear implantation (101.02 ± 7.43 and 94.71 ± 8.06, respectively). Based on these data, we concluded that a score difference of 7.3 (the standard deviation of the two groups was 7.7) between different groups had a clinical implication. Considering that the standard deviation of the two groups was 7.7, *α* = .05 and 1‐*β* = .80, our study needed 25 participants in each group. We enrolled all possible participants during the study period to ensure that the group with the smallest number of participants also met the analysis requirements.

### 2.7 Statistical analysis

Statistical analyses were performed using SAS 9.4 (SAS Institute, Inc.). Continuous data were expressed as means ± standard deviations, while categorical data were expressed as frequencies and percentages. Descriptive statistics were used to summarize the patients’ baseline characteristics. Between‐group comparisons of quantitative and categorical data were performed using independent sample *t*‐tests or Mann–Whitney tests and Cochran–Mantel–Haenszel χ^2^ tests, respectively. Continuous data were evaluated using a two‐tailed independent *t*‐test or Wilcoxon's rank sum test, while categorical data were evaluated using a two‐tailed chi‐square or Wilcoxon's rank sum test. A multiple linear regression model was used to adjust for covariates. Statistical significance was set at *p* < .05.

## 3 RESULTS

### 3.1 Demographics and clinical characteristics

**FIGURE 1:**
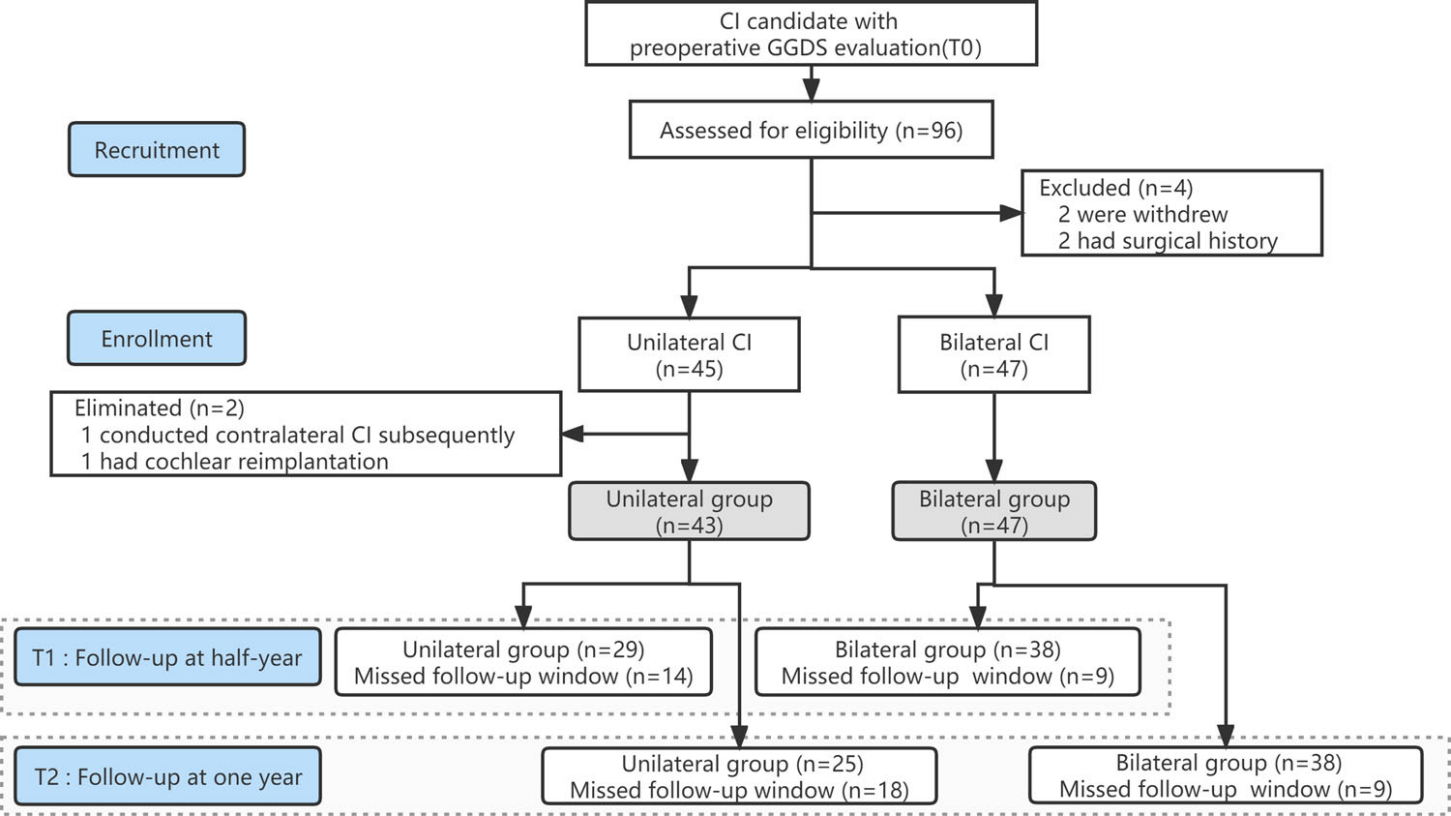
Flow diagram of the study.

Among the 96 screened participants, six were excluded for violation of the research design (Figure 1). Finally, 90 patients (46 males and 44 females) were enrolled in this study; among them, 43 (48%) and 47 (52%) patients underwent unilateral and bilateral cochlear implantation, respectively (Figure 1).

Table 1 shows the characteristics and the baseline score of the participants in each group. The unilateral group had a significantly higher mean age than the bilateral group (15.63 ± 6.99 vs. 11.00 ± 3.66 months, respectively; *p =* .001), as well as a higher mean weight and height. The percentage of those wearing hearing aids was significantly higher in the unilateral group than the bilateral group (79.07% vs. 34.04%, *p* < .001). The surgery and anesthesia durations were significantly shorter in the unilateral group than the bilateral group (125.26 ± 25.88 vs. 250.40 ± 36.77 min and 148.81 ± 25.60 vs. 271.57 ± 36.09 min, respectively; *p* < .001).

**TABLE 1:** Characteristics of each group.

Items	Unilateral group (*n* = 43)	Bilateral group (*n* = 47)	*p*‐Value
Demographic characteristics			
Sex, male (%)	26 (60.47%)	20 (42.55%)	.090
Age (months)	15.63 ± 6.99	11.00 ± 3.66	<**.001**
Weight (kg)	10.75 ± 2.06	9.60 ± 1.07	**.001**
Height (cm)	80.87 ± 8.11	74.20 ± 4.20	<**.001**
Hearing aid (yes, %)	34 (79.07%)	16 (34.04%)	<**.001**
Surgery dimensions			
Surgical time (min)	125.26 ± 25.88	250.40 ± 36.77	<**.001**
Anesthesia duration (min)	148.81 ± 25.60	271.57 ± 36.09	<**.001**
Midazolam (mg kg^−1^)	0.06 ± 0.01	0.05 ± 0.01	.522
Fentanyl (μg kg^−1^)	3.83 ± 1.32	4.94 ± 1.65	<**.001**
Propofol (mg kg^−1^ h^−1^)	4.83 ± 1.26	4.48 ± 1.38	.104
Remifentanil (μg kg^−1^ min^−1^)	0.12 ± 0.04	0.13 ± 0.04	.060
Dexmedetomidine (μg kg^−1^ h^−1^)	0.50 ± 0.26	0.51 ± 0.26	.436
Total propofol (mg)	125.19 ± 33.46	192.35 ± 60.79	<**.001**
Total remifentanil (μg)	0.18 ± 0.05	0.34 ± 0.11	<**.001**
Total dexmedetomidine (μg)	12.83 ± 5.95	22.22 ± 12.41	<**.001**
Mean scores for each GDDS domain at T0			
Gross motor	106.52 ± 13.17	101.77 ± 8.65	**.045**
Fine motor	99.37 ± 8.07	101.46 ± 7.86	.218
Adaptability	100.29 ± 9.42	98.47 ± 7.51	.312
Language	58.74 ± 20.76	53.53 ± 16.12	.185
Social skills	94.88 ± 11.08	96.39 ± 7.53	.447

Abbreviations: DQ, developmental quotient; GDDS, Gesell Development Diagnosis Scale. blod markers the *p* value less than 0.05.

Regarding the baseline developmental levels, no significant between‐group differences were found in the fine motor, adaptability, language, and social skills at baseline. The language DQs of participants were both <85, which implied that the participants did not reach a “Normal” level of language (DQ ≥85 was regarded as “Normal,” according to the clinical diagnostic criteria). However, the gross motor DQ was higher in the unilateral group than the bilateral group (106.52 ± 13.17 vs. 101.77 ± 8.65, *p =* .045) (Table 1).

### 3.2 Follow‐up

**FIGURE 2:**
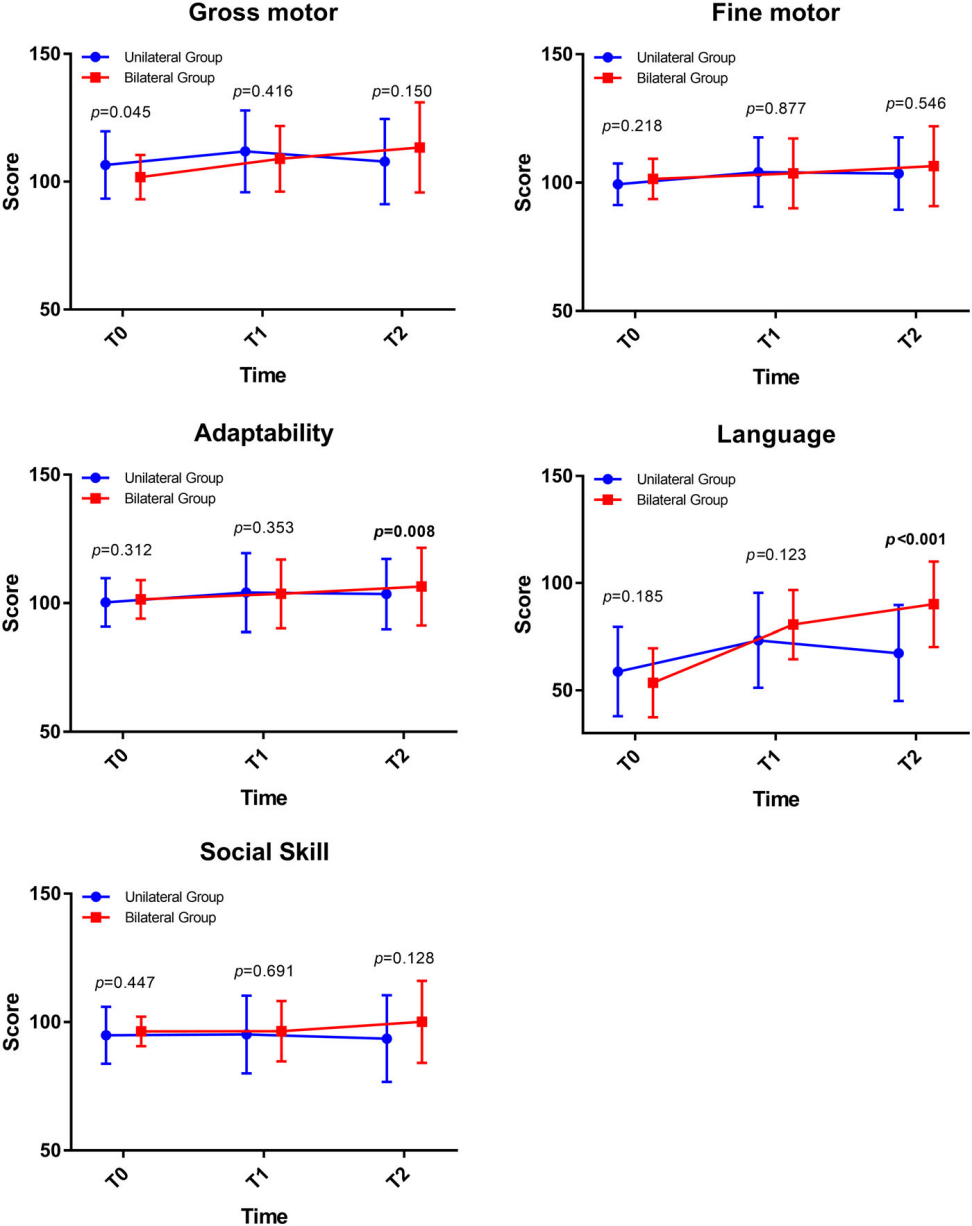
The Gesell Development Diagnosis Scale (GDDS) results in the unilateral and bilateral groups over time in the domains of gross motor, fine motor, adaptability (*p =* .008), language (*p* < .001), and social skills.

There were 29 and 38 patients in the unilateral and bilateral groups, respectively, who completed the T1 follow‐up, while 25 and 38 patients in the unilateral and bilateral groups, respectively, completed the T2 follow‐up within the preset time windows (Figure 1). Additionally, no significant between‐group difference was observed in the fine motor scale at T1 or any other endpoint at T1. Notably, significant between‐group differences were found in the adaptability and language domains at T2 (Figure 2).

The bilateral group presented better trends in adaptability and language than the unilateral group. Therefore, the *t*‐test was used for between‐group comparisons of the DQs at each time point. The DQs for adaptability and language domains were higher in the bilateral group than the unilateral group at 1 year (*p =* .008 and *p* < .001, respectively). It showed a clinically significant difference in the language domain; the proportions of normal, suspicious, and delay were 65.78%, 26.31%, and 7.89% in the bilateral group at T2, respectively, while the proportions of normal, suspicious, and delay were 16%, 20%, and 64% in the unilateral group, respectively. No significant between‐group differences were found in the DQs for gross motor, fine motor, and social skill domains (Figure 2).

We performed between‐group comparisons of changes in the GDDS scores from baseline to T1 and T2 with adjustment for variables at baseline in a crude model (Model 1) or further adjusted, including baseline results, age, sex, and preoperative hearing aid use as covariates (Model 2). In both models, the changes in the DQs for the language domain were significantly larger in the bilateral group than the unilateral group at T1 (25.07 ± 4.37 vs. 10.88 ± 4.61 in Model 1, *p* < .001; 25.44 ± 4.93 vs. 10.39 ± 5.72 in Model 2, *p* < .001) and T2 (34.98 ± 5.94 vs. 15.28 ± 6.55 in Model 1, *p* < .001; 35.34 ± 6.37 vs. 12.99 ± 7.83 in Model 2, *p* < .001). Regarding the adaptability domain, slight improvement at T2 was observed in the bilateral group in Model 1 (14.00 ± 4.54 vs. 5.82 ± 5.01, *p =* .019) but not in Model 2 (12.55 ± 4.27 vs. 6.78 ± 5.29, *p =* .128) (Table 2).

**TABLE 2:** Changes in the Gesell Development Diagnosis Scale (GDDS) score from baseline to T1 and T2_._

Items	Change from baseline		Difference (95% CI)	*p*‐Value
	Unilateral group (95% CI)	Bilateral group (95% CI)		
Model 1				
T1 N (missing)	29 (14)	38 (9)		
Gross motor	5.58 (1.05–10.11)	6.95 (2.66–11.24)	−1.37 (−7.69 to 4.95)	.667
Fine motor	3.50 (−0.81 to 7.81)	2.67 (−1.42 to 6.75)	0.83 (−5.14 to 6.80)	.783
Adaptability	5.32 (0.68–9.96)	8.74 (4.34–13.14)	−3.42 (−9.84 to 2.99)	.291
Language	10.88 (6.27–15.49)	25.07 (20.70–29.44)	−14.19 (−20.60 to −7.78)	<**.001**
Social skill	−2.70 (−6.67 to 1.26)	0.50 (−3.26 to 4.26)	−3.20 (−8.65 to 2.27)	.247
T2 N (missing)	25 (18)	38 (9)		
Gross motor	4.93 (−1.16 to 11.01)	11.08 (5.57–16.59)	−6.15 (−14.49 to 2.18)	.146
Fine motor	5.63 (0.04–11.23)	6.83 (1.76–11.91)	−1.20 (−8.84 to 6.44)	.755
Adaptability	5.82 (0.81–10.82)	14.00 (9.46–18.54)	−8.18 (−14.96 to −1.40)	**.019**
Language	15.28 (8.73–21.83)	34.98 (29.04–40.92)	−19.70 (−28.60 to −10.80)	<**.001**
Social skill	0.35 (−4.90 to 5.60)	4.85 (0.08–9.62)	−4.50 (−11.59 to 2.59)	0.210
Model 2				
T1 N (missing)	29 (14)	38 (9)		
Gross motor	5.68 (0.28–11.08)	6.38 (1.79–10.98)	−0.70 (−8.54 to 7.13)	0.859
Fine motor	3.02 (−2.27 to 8.32)	2.73 (−1.77 to 7.24)	0.29 (−7.41 to 7.99)	.940
Adaptability	3.58 (−1.80 to 8.96)	9.00 (4.43–13.58)	−5.42 (−13.22 to 2.38)	.170
Language	10.39 (4.67–16.11)	25.44 (20.51–30.36)	−15.05 (−23.44 to −6.66)	<**.001**
Social skill	−3.45 (−8.21 to 1.30)	0.44 (−3.60 to 4.48)	−3.89 (−10.80 to 3.02)	.265
T2 N (missing)	25 (18)	38 (9)		
Gross motor	5.23 (−1.73 to 12.20)	9.91 (4.28–15.54)	−4.68 (−14.53 to 5.17)	.346
Fine motor	6.17 (−0.46 to 12.79)	6.13 (0.76–11.50)	0.04 (−9.34 to 9.41)	.994
Adaptability	6.78 (1.49–12.08)	12.55 (8.28–16.82)	−5.77 (−13.23 to 1.69)	.128
Language	12.99 (5.16–20.82)	35.34 (28.97–41.71)	−22.35 (−33.48 to −11.22)	<**.001**
Social skill	0.64 (−5.53 to 6.80)	4.29 (−0.69 to 9.27)	−3.65 (−12.36 to 5.06)	.406

*Note*: Model 1: The only adjusted variable is the baseline result. Model 2: Adjusted variables include baseline result, age, sex, and preoperative hearing aid.

Abbreviations: T1, half‐year follow‐up; T2, one‐year follow‐up; CI, confidence interval.

**FIGURE 3:**
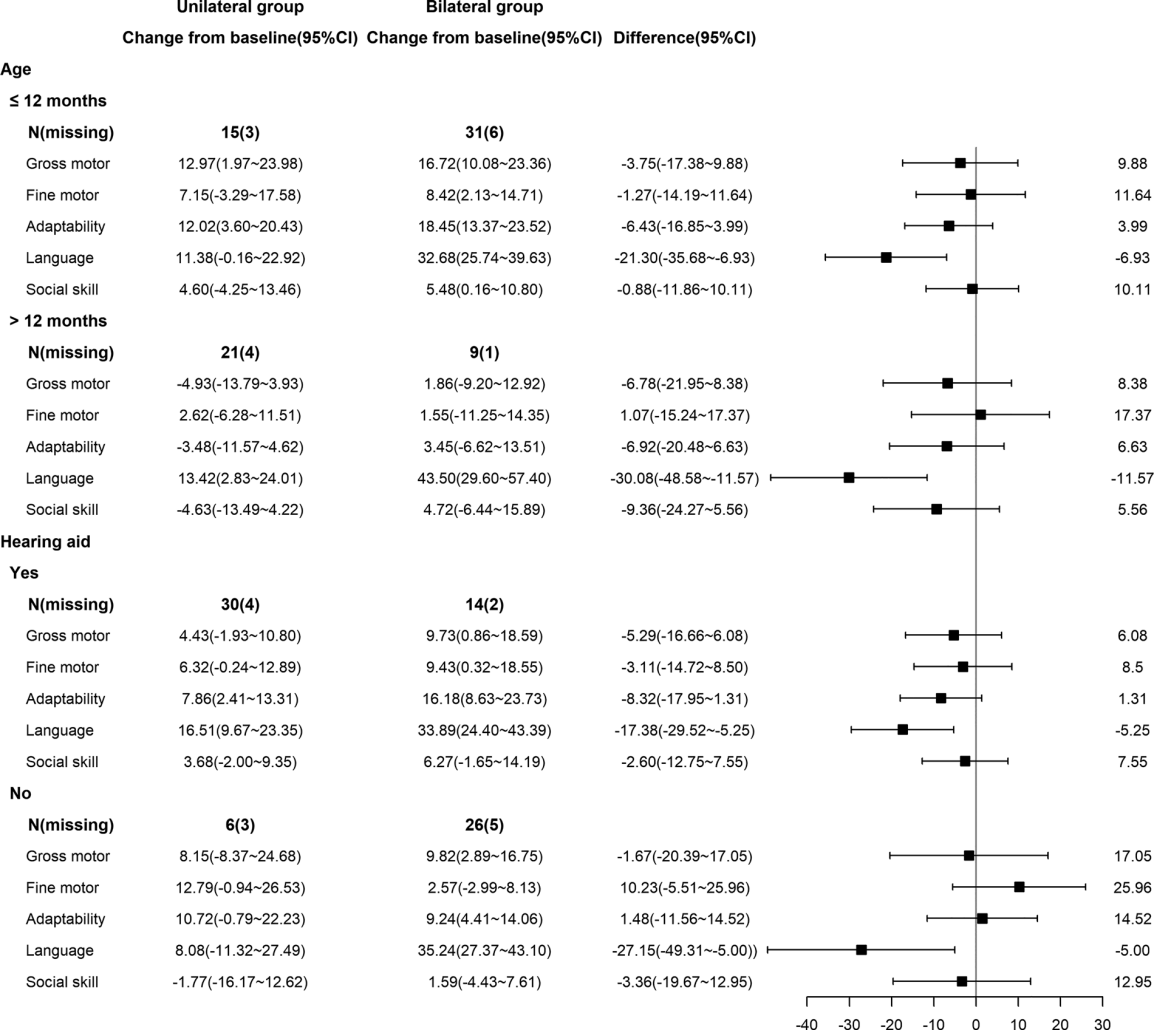
The Gesell Development Diagnosis Scale (GDDS) results in the unilateral and bilateral groups at 1 year after cochlear implantation, stratified by age and preoperative hearing aid use.

Due to the baseline differences in preoperative age and wearing of hearing aids between the two groups, we showed the joint distribution of the following two binary sub‐grouping variables within the 1‐year follow‐up: age ≤12 months/age > 12 months and preoperative hearing aid/non‐preoperative hearing aid (Figure 3). After stratifying according to age, significant improvement was observed only in the language domain in the bilateral group compared to the unilateral group at T2 (*p =* .005 and *p =* .003, respectively). Moreover, after stratifying according to preoperative hearing aid use, the bilateral group showed significantly better DQs for the language domain than the unilateral group at T2, both among patients with (*p =* .06) and without hearing aids (*p =* .018).

Furthermore, we constructed stepwise linear regression models to explore factors that influenced each domain in both groups (Table 3). The gross and fine motor DQs at T1 were positively correlated with those at T0 (*β* = .354, *p =* .025 and *β* = .428, *p =* .027, respectively). The language DQs at T1 were positively correlated with those at T0 (*β* = .684, *p* < .001) and the treatment group (bilateral) (*β* = 13.249, *p =* .001). The social skill DQs at T1 were positively correlated with those at T0 (*β* = .506, *p =* .002) and negatively correlated with the weight at T0 (*β* = −1.998, *p =* .047).

**TABLE 3:** The multiple regression of factors influencing each domain developmental quotient (DQ) of Gesell Development Diagnosis Scale (GDDS) at T1 and T2.

Follow‐up	Items	Variable in final model after stepwise	*β*	SE	*t*	*p*
T1						
	Gross motor					
		Gross motor in T0	0.354	0.149	2.38	.025
	Fine motor					
		Fine motor in T0	0.428	0.189	2.26	.027
	Language					
		Language in T0	0.684	0.099	6.91	<.001
		Treatment group	13.249	3.915	3.38	.001
	Social skill					
		Social skills in T0	0.506	0.159	3.18	.002
		Weight	−1.998	0.985	−2.03	.047
T2						
	Gross motor					
		Age	−1.106	0.453	−2.44	.018
	Adaptability					
		Age	−1.639	0.354	−4.63	<.001
	Language					
		Language in T0	0.641	0.145	4.42	<.001
		Treatment group	20.998	5.339	3.93	<.001
	Social skill					
		Age	−1.132	0.411	−2.75	.008

Abbreviations: T0, Pre‐operation; T1, half‐year follow‐up; T2, 1‐year follow‐up.

At 1 year after cochlear implant activation, age was negatively correlated with the gross motor, fine motor, and social skill DQs at T2. However, language DQs at T2 remained positively correlated with those at T0 (*β* = .641, *p* < .001) and the treatment group (bilateral) (*β* = 20.998, *p* < .001).

## 4 DISCUSSION

In this observational cohort study, there was no evidence of a significant difference that exposure to prolonged anesthesia produced negative neurological outcomes in children with hearing impairment. In contrast, our study showed that early intervention for bilateral cochlear implantation improves language DQ and promotes the development of gross motor, adaptability, and social skill DQs, although the participants were exposed to longer periods of anesthesia at a younger age. The results may also be of interest to otolaryngologists.

This study enrolled children with bilateral deafness who had normal development as participants to reduce confounding factors among the research participants and avoid interference with subsequent follow‐up. According to the current international guidelines of clinical protocols and candidacy recommendation (Society of Otolaryngology C M A, 2003; Warner‐Czyz et al., 2022), cochlear implantation should be considered for children with prelingual bilateral SNHL when hearing aids do not enable language development. Professionals and families should consider audiometric criteria, speech perception, functional outcomes, residual hearing, and economic factors when referring a child for cochlear implantation candidacy evaluation (Warner‐Czyz et al., 2022). Although several factors contribute to the age at implantation, including the advent, acceptance, and implementation of early hearing detection and intervention programs and subsequent early initial hearing aid fitting (Society of Otolaryngology C M A, 2003), early cochlear implantation provides children with hearing disability the best chance to close the spoken language gap with their hearing peers (Warner‐Czyz et al., 2022). Hearing disability may influence overall development in the auditory cortex, assuming other functions in the case of no interventions (Mckay, 2018).

In our study, the unilateral group had higher mean age, weight, and height, as well as a higher rate of use of preoperative hearing aids than the bilateral group. The parents of patients who wear hearing aids and undergo hearing rehabilitation training may seek interventions at a later age. The possible cause of the better development of preoperative gross motor skills in the unilateral group may be due to brain plasticity resulting from the residual hearing. Furthermore, 1 year after cochlear implantation activation, the gross motor skills in the bilateral group had a faster growth trend, although no statistical difference was found between the two groups.

The duration of surgery and anesthesia was longer in the bilateral group than in the unilateral group. However, no between‐group difference was observed in the maintenance dose of propofol, remifentanil, and dexmedetomidine per kilogram of body weight per hour/minute. Screening of the perioperative medical records revealed that anesthesiologists administered an additional medication bolus of fentanyl, rather than a maintenance anesthetic, usually before cochlear implantation on the second side to reduce cardiovascular stress. Specifically, both groups received the same anesthetic regimen, with only the anesthesia duration differing. Therefore, prescinding the benefits of cochlear implantation, no evidence of a significant difference existed in developmental impairment induced by a long (>3 h) and short (<3 h) anesthesia duration.

The GDDS, developed by Dr. Arnold Gesell at Yale University (Gesell & Amatruda, 1941) as a child development test scale, is used to measure mental and psychological development in normal infants and young children; moreover, it is used as a diagnostic tool for those with neurological or mental disorders (Youm et al., 2013). Chen et al. (2023) showed that the GDDS had good sensitivity in evaluating the short‐term effects of bilateral cochlear implants on neuropsychological development and enabled reliable prediction of speech production among very young cochlear implant users. Yang et al. (2017) and Meinzen‐Derr et al. (2017) reported that preoperative GDDS scores could reliably predict subsequent early‐childhood development for pediatric cochlear implant recipients.

In our study, the gross motor, adaptability, and social skill DQs at T2 were negatively correlated with the age at operation. Chen et al. (2023) performed simultaneous bilateral cochlear implant surgery in young children who experienced similar long‐duration anesthesia and reported that the age at bilateral cochlear implantation was negatively correlated with the DQs of language, social skills, and adaptability in children after 2 years postoperatively. This conclusion was similar to our research results. In our study, we observed no between‐group differences in the improvement of each domain at the half‐year follow‐up, except for the language DQ. The average language DQs between‐group were not different (53.53 ± 16.13 and 58.74 ± 20.63 in the bilateral and unilateral groups, respectively) at T0. According to the clinical diagnostic criteria, both groups were identified as having significant developmental delays with DQ ≤75. The language DQs in the bilateral group at T2 improved by approximately 40, implying that the language DQ reached the level of normal neurological development, as defined by DQ ≥86. However, the unilateral group only improved by ∼15 and had not yet reached normal development levels at T2. Furthermore, the language DQs at T1 were positively correlated with those at baseline; moreover, the bilateral group had higher language DQs at T1 and T2 than the unilateral group. Similar findings were observed at the 1‐year follow‐up and were further validated in the subgroup analysis according to age and preoperative use of hearing aids. Specifically, the bilateral group showed a more significant improvement in language DQs than the unilateral group, regardless of the age at implantation. Exploratory multiple linear regression analysis showed that the earlier the cochlear implantation, the greater the improvement of gross motor, adaptability, and social skill DQs at 1 year postoperatively. It suggests that exposure to prolonged anesthesia during bilateral cochlear implantation does not affect cognitive function in infants with SNHL.

Our previous study in young mice, which received multiple 2.5% sevoflurane anesthesia, showed that the expression of the presynaptic marker, synaptophysin, in the prefrontal cortex was reduced and caused a deficit in fine motor, learning, and memory functions. Coincidentally, in infants who underwent a total inhalation anesthesia regimen with 2.0%−3.5% sevoflurane maintenance for longer than 5 h, a slight reduction was observed in fine motor DQ at 6 months postoperatively (Zhang et al., 2022). Currently, we prefer a lower concentration of inhaled anesthetic to facilitate the electromyography of facial nerve monitor in cochlear implantation surgery. The difference between these two studies could be attributed to the mix‐agent general anesthetic regimen used to reduce the dosage of sevoflurane, which might impair cognitive functions at high minimum alveolar concentrations. A sibling‐matched cohort study reported that the healthy children with a single anesthesia exposure before the age of 36 months, compared with those with no anesthesia exposure, had no significant differences in intelligence quotient scores in later childhood (Sun et al., 2016). However, children with multiple early‐life exposures to anesthesia, not with a single exposure, had poor scores found in parent‐reported behavior (Warner et al., 2019), learning difficulties (Warner et al., 2018), processing speed, and executive function (Zaccariello et al., 2019). The American Society of Anesthesiologists Physical Status III–IV proportion (18.9%) was higher in the multiple anesthesia exposure group than the single exposure group (2.4%). Since anesthesia and surgery occur concomitantly, it remains unclear whether the potential cognitive impairment is attributable to the use of anesthetics or other unobserved factors. These trials suggested that prolonged anesthetic exposure in otherwise healthy children had limited effects. Nevertheless, concerns regarding anesthetic neurotoxicity are myriad and nuanced. Each drug has some undesirable adverse effects that, together with the patient's medical and surgical history, influence the choice of the most suitable anesthetic agent for a specific situation. Several studies have reported little or no effects of anesthetic exposure in children on the intelligence quotient, academic achievement, and teacher evaluations. Therefore, although controversy exists regarding the effect of anesthetic exposure on neurodevelopment in children, there is a consensus that it does not cause significant deficits across all cognitive domains (Ing & Brambrink, 2019), which is consistent with our findings.

This study had some limitations. First, this was a single‐center study, which limits the generalizability of these results, given the lack of diversity in the study population. Therefore, multicenter studies are warranted to validate our findings. Second, significant between‐group differences were observed in the ages of the participants. Although we adjusted for these variables, the confounding effects may not have been sufficiently eliminated since the use of hearing aids in the unilateral group may stimulate residual hearing and may have improved their overall development. These differences cannot be avoided in an observational study. However, subgroup analyses revealed no interaction between the hearing aid and group effects. Third, confounding factors may have influenced the cochlear implantation outcomes, such as household income levels and parental education and the rehabilitation training after cochlear implantation. Each participant received speech rehabilitation offered by different speech‐language pathologists in local organizations affiliated with the Disabled Persons’ Federation, and that would affect the outcomes and GGDS scores. It was impossible to homogenize the participants in this observational study. Finally, limited evaluation tools are available in clinical situations for infants with hearing impairment. We used the GDDS only for development evaluation, and the participants were followed up 1 year after cochlear implant activation. Due to the coronavirus disease 2019 pandemic, the missed follow‐up rate was high. The final sample size may have been insufficient to draw definitive conclusions regarding long‐term anesthetic‐induced developmental impairment.

## 5 CONCLUSION

Prolonged anesthetic exposure does not associate with developmental impairment, as measured using the GDDS, in infants undergoing bilateral cochlear implantation. Additionally, compared with unilateral cochlear implantation, bilateral cochlear implantation in early life significantly improves language ability and promotes the development of adaptability. Further large‐scale studies with long follow‐up periods are warranted to validate our findings.

## AUTHOR CONTRIBUTIONS

Project administration: Jingjie Li. Visualization: Yue Yu. Validation: Xuhui Zhou. Software: Nanyang Le, Yudan Liang. Supervision: Hong Jiang. Roles/Writing: Original Draft: Jinya Shi, Li Ma. All authors have read and approved the final manuscript.

## CONFLICT OF INTEREST STATEMENT

The authors declare no conflict of interest.

### ETHICS STATEMENT

This study was approved by the Human Research Ethical Committee of Shanghai Ninth People's Hospital, Shanghai JiaoTong University of Medicine, P. R. China (SH9H‐2019‐T19‐2) and registered at ClinicalTrials.gov (NCT04255485).

### PEER REVIEW

The peer review history for this article is available at https://publons.com/publon/10.1002/brb3.3216.

## Data Availability

The data that support the findings of this study are available from the corresponding author upon reasonable request.
